# The Burgeoning Significance of Liquid-Liquid Phase Separation in the Pathogenesis and Therapeutics of Cancers

**DOI:** 10.7150/ijbs.92988

**Published:** 2024-02-12

**Authors:** Wei-Xin Xu, Qiang Qu, Hai-Hui Zhuang, Xin-Qi Teng, Yi-Wen Wei, Jian Luo, Ying-Huan Dai, Jian Qu

**Affiliations:** 1 Department of Pharmacy, the Second Xiangya Hospital, Central South University; Institute of Clinical Pharmacy, Central South University, Changsha 410011, China.; 2 Department of Pharmacy, Xiangya Hospital, Central South University, Changsha 410078, China.; 3 National Clinical Research Center for Geriatric Disorders, Xiangya Hospital, Central South University, Changsha 410078, China.; 4 Department of Pathology, the Second Xiangya Hospital, Central South University, Changsha 410011, China.; 5 Hunan key laboratory of the research and development of novel pharmaceutical preparations, Changsha Medical University, Changsha 410219, China.

**Keywords:** liquid-liquid phase separation, biomolecular condensates, cancer, super enhancer, disease

## Abstract

Liquid-liquid phase separation (LLPS) is a physiological phenomenon that parallels the mixing of oil and water, giving rise to compartments with diverse physical properties. Biomolecular condensates, arising from LLPS, serve as critical regulators of gene expression and control, with a particular significance in the context of malignant tumors. Recent investigations have unveiled the intimate connection between LLPS and cancer, a nexus that profoundly impacts various facets of cancer progression, including DNA repair, transcriptional regulation, oncogene expression, and the formation of critical membraneless organelles within the cancer microenvironment. This review provides a comprehensive account of the evolution of LLPS from the molecular to the pathological level. We explore the mechanisms by through which biomolecular condensates govern diverse cellular physiological processes, encompassing gene expression, transcriptional control, signal transduction, and responses to environmental stressors. Furthermore, we concentrate on potential therapeutic targets and the development of small-molecule inhibitors associated with LLPS in prevalent clinical malignancies. Understanding the role of LLPS and its interplay within the tumor milieu holds promise for enhancing cancer treatment strategies, particularly in overcoming drug resistance challenges. These insights offer innovative perspectives and support for advancing cancer therapy.

## Introduction

In both human society and business, the effective collaboration of diverse groups often necessitates their organization into functional systems. A parallel concept exists within the realm of cellular biology, where human cells employ a process known as liquid-liquid phase separation (LLPS) to partition their constituents. This in vivo physiological mechanism is believed to underlie the formation of membrane-free structures within cells, such as nucleoli 1. The resulting regionalization of cellular activities, often referred to as "biomolecular condensates," arises from the coalescence of various components of membraneless cellular structures via LLPS 2. These condensates play key roles in numerous cellular functions, including the regulation of gene expression, control of nucleic acid transcription, and in vivo responses to environmental stimuli 2, 3. This physiological phenomenon, LLPS, has gained considerable attention in recent research 1. It is integral to our understanding of how cells compartmentalize and optimize their functions. Previous research has extensively investigated the formation of condensates in the context of amyotrophic lateral sclerosis 4. As our comprehension of LLPS continues to evolve, pathological processes related to neurodegenerative diseases and certain cancers are also being viewed anew as manifestations of LLPS-driven processes 5, 6. A profound grasp of these biomolecular condensates assumes paramount importance in the exploration of diverse biological phenomena and the formulation of therapeutic interventions.

This comprehensive review aims to elucidate the physiological and biochemical underpinnings of LLPS and biomolecular condensates, thereby facilitating a more profound comprehension of the intricate relationship between LLPS and cancer. Our focus centers on elucidating the mechanisms through which phase separation orchestrates the progression of select malignancies, offering valuable insights into the realm of targeted therapies and strategies to overcome drug resistance challenges. By delving into these intricacies, this review endeavors to provide researchers with a robust foundation for further inquiry and advancement in the field.

## Development of LLPS

### Development History

**Figure 1:**
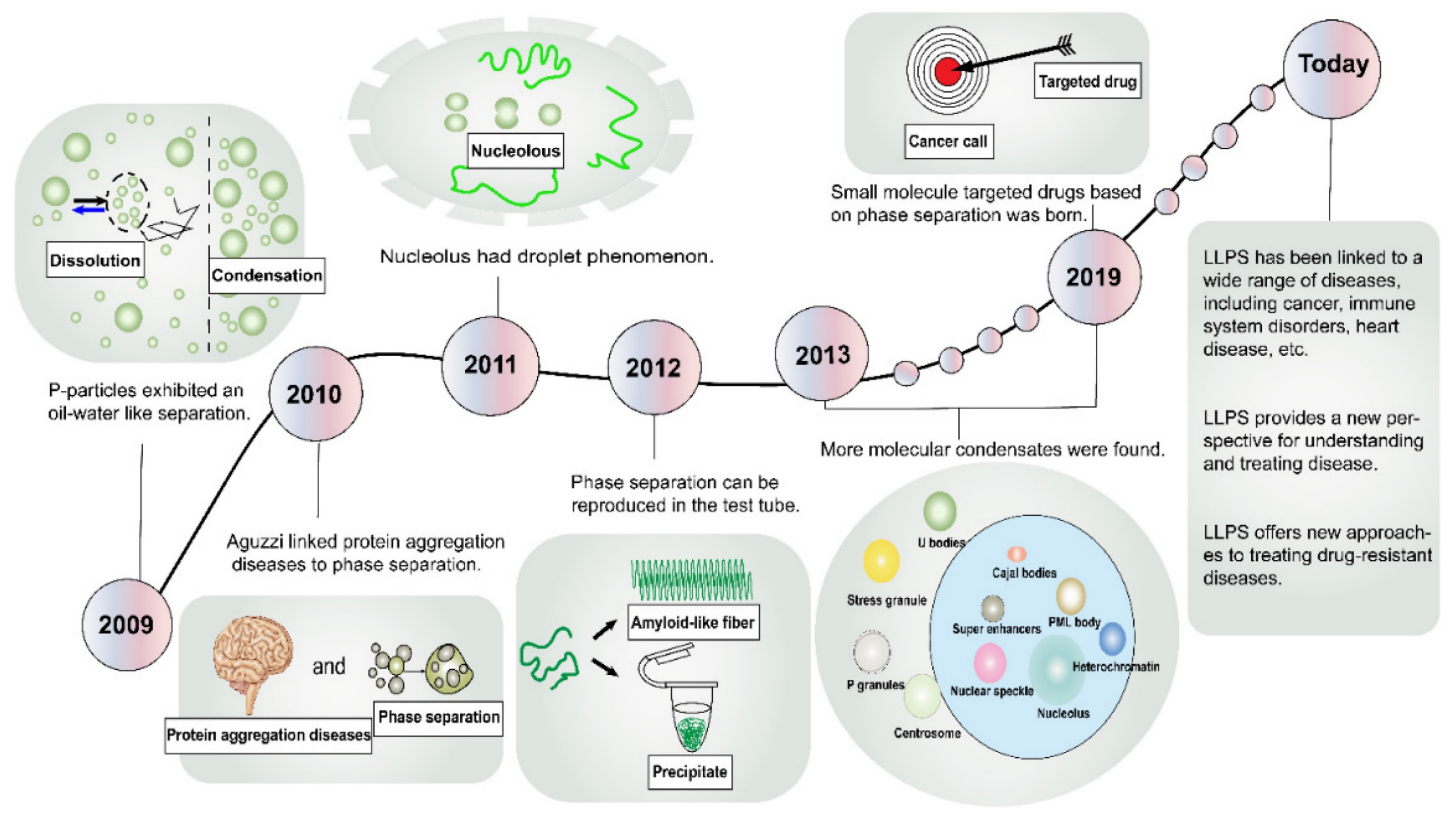
History of the discovery and development of LLPS. Representative milestone findings promoting the development of LLPS are enumerated in the figure. LLPS, liquid-liquid phase separation; PML, promyelocytic leukemia.

In the domain of physical chemistry, the term “phase separation” conventionally denotes the spontaneous demixing of two coexisting fluids 7. Remarkably, the literature has harbored descriptions of membraneless organelles for over a century, yet within the biological community, few researchers have paid them much heed 8. A critical turning point transpired in 2009 when Brangwynne et al. discerned “oil-water” separation-like behaviors within P granules in Caenorhabditis elegans (**Figure 1**) 9. Subsequent to this, in 2010, Aguzzi et al. probed the pathogenesis of protein aggregation diseases, revealing an intimate linkage to phase separation dynamics 10. In 2011, Brangwynne et al. extended their explorations to nucleosomes, uncovering analogous fluidic traits 11. The year 2012 witnessed Li and Kato et al. harnessing molecular methodologies to simulate phase separation phenomena within the laboratory setting 12, 13. Subsequent endeavors unearthed an array of molecular condensates, encompassing Cajal bodies 14, nuclear speckles 15, stress granules (SGs) 16, signaling puncta 17, promyelocytic leukemia (PML) bodies 18, super-enhancers (SEs) 19, heterochromatin 20, and more. These advancements in cell structure elucidation have precipitated innovative avenues for disease intervention. In a landmark development in 2019, Wheeler et al. identified lipoamide as the first small molecule capable of targeting the phase separation of the fused-in sarcoma (FUS) protein, thereby paving the way for the treatment of neurodegenerative disorders 21. In recent years, the frontiers of LLPS research have expanded exponentially, culminating in a burgeoning body of literature linking LLPS to a spectrum of diseases, including cancer, infectious ailments, cardiovascular disorders, and neurological maladies 22-25.

### Formation Mechanisms of LLPS

The fundamental physical principles that underpin the phenomenon of LLPS in polymers have gained considerable attention and have shed light on the understanding of LLPS in biological systems 26. The concentration of molecules within a limited space involves an energy cost, yet numerous weak interactions can offset the entropic cost associated with LLPS 27.

LLPS is thought to be initiated by weak, multivalent interactions, often provided by intrinsically disordered regions and structured domains in proteins, which aid in specific molecular recognition 28. For example, aromatic residues such as tyrosine, with their side chains containing delocalized π electrons, contribute to π-π stacking interactions that facilitate LLPS 29. Notably, it has been observed that π-π interactions are also present in non-aromatic amino acids, as demonstrated in the fragile X mental retardation protein 29. Positively charged amino acids, such as lysine and arginine, participate in cation-π interactions with electron-rich aromatic groups, further promoting LLPS 30. Interestingly, these cation-π interactions are strong enough to overcome the repulsive effects typically seen in cation-anion interactions, thus aiding in phase separation among similarly charged molecules 31. Charge-charge interactions are increasingly recognized as key contributors to LLPS. The aggregation of polymers with opposing charges, resulting in charge neutralization, can lead to the formation of droplets, as observed in mixtures of RNA and cationic peptides 32, 33. Moreover, hydrophobic interactions and dipole-dipole interactions, which are independent of the amino acid composition of low complexity domains, have been suggested as mechanisms driving LLPS 26, 34.

The role of LLPS in the regulation of cellular responses is a highly intricate and precisely controlled process, involving not only weak intermolecular interactions within cells but also significantly influenced by intra- and extracellular environmental factors. Key environmental variables include temperature, ion concentrations, and pH levels, each capable of modulating the physicochemical properties of cellular molecules, thereby impacting the occurrence and characteristics of LLPS 35-37. Additionally, in certain pathological states, such as some neurological disorders, the aberrant aggregation of proteins is closely associated with alterations in the LLPS process, suggesting a potential role of this mechanism in disease onset and progression 38. The energy state of the cell, particularly ATP levels, also plays a crucial role in influencing the dynamics of LLPS 39. Overall, LLPS as a mechanism for regulating cellular responses relies on the interplay of multiple protein interactions and a nuanced response to the cellular environment. This dynamic separation mechanism contributes to the organization and functional regulation of intracellular spaces, ensuring the optimal execution of cellular responses.

### Physiological Functions of LLPS

**Figure 2:**
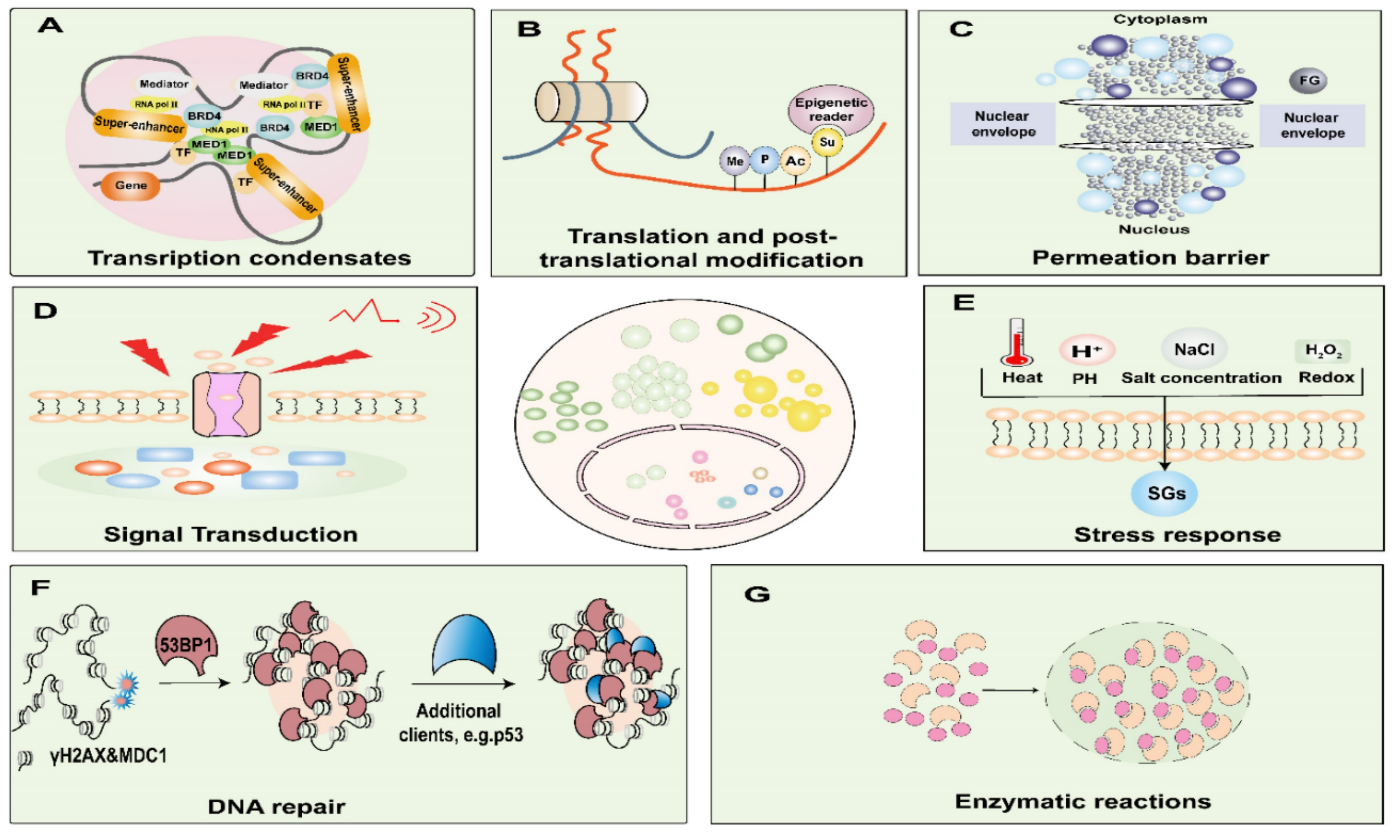
Functions of biomolecular condensates. Biomolecular condensates are involved in nuclear functions and are critical for DNA damage, nuclear translocation, enzymatic reactions, amplification, transcription, translation, and post-translational modification processes. Condensates are involved in signal transduction, stress sensing, and transport processes. A. Model of a phases-separated complex at gene regulatory elements. At the super-enhancer locus, transcriptional regulators with extensive interactions, including TFs, BRD4, MED1, and RNA pol II, are enriched to form a phase-separated condensate, which is separated from others. B. Post-translational modification processes such as phosphorylation, acetylation, ubiquitination, and SUMOylation. C. Among the FG-nucleoporins form the central pore of the nuclear pore complexes, which governs the nucleocytoplasmic transport through the pores. D. Condensates are involved in signal transduction. E. Condensates are involved in stress response. F. After DNA damage sites cause the formation of γH2AX and recruitment of MDC1 for nucleation, 53BP1 accumulates and phase separates, and *P53* acts as a scaffold for client molecules, interacting transiently with 53BP1, where they find an environment permissive for their activation. G. Phase separation significantly accelerates the efficiency of multienzyme biocatalysis. BRD4, bromodomain-containing protein; RNA Pol II, RNA polymerase II; MED1, mediator complex subunit 1; TF, transcription factors. Me, methylation; P, phosphorylation; Ac, acetylation; Su, and small ubiquitin-like modifier; FG, Phenylalanine-glycine-rich; SGs, stress granules; 53PB1, *p53* binding protein 1; γH2AX, phosphorylated histone H2AX; MDC1, Mediator of DNA damage checkpoint protein 1.

LLPS exerts intricate regulatory control over a spectrum of biological processes and functions, encompassing but not limited to gene expression, signal transduction, enzymatic responses, and stress response (**Figure 2**) 40. It is imperative to acknowledge that the aberrant occurrence of LLPS, either temporally or spatially, has the potential to culminate in the formation of recalcitrant and irreversibly structured entities. Such untoward events can precipitate obstructive phenomena or pathological aggregations, thereby underpinning the onset of various maladies 41. Consequently, a comprehensive comprehension of the molecular underpinnings of LLPS in the context of cancer assumes paramount significance, constituting a critical prerequisite for the development of efficacious therapeutic strategies.

### Involvement in the function of the nucleus

#### LLPS in transcriptional regulation

Transcription factors (TFs) exert meticulous control over gene expression by virtue of their ability to bind to cis-acting regulatory elements, known as enhancers, and by orchestrating the recruitment of coactivators alongside RNA polymerase II (Pol II) 42. Remarkably, SEs, comprised of several hundred clusters of enhancers, wield the authority to initiate transcription of vital genes that underpin cell identity, thanks to the phase-separating attributes residing within the intrinsically disordered regions of TFs and cofactors 43. Emerging evidence has illuminated the key role of phase-separated condensates in steering transcriptional regulation 44. Notably, TFs such as the Octamer-binding transcription factor 4 (OCT4) governing embryonic stem cell pluripotency and the yeast TF GCN4 have been shown to form phase-separated condensates alongside Mediator, an indispensable coactivator complex 45. Sabari et al. have eloquently demonstrated that transcriptional coactivators enriched in SEs, namely, bromodomain-containing protein 4 (BRD4) and mediator complex subunit 1 (MED1), manifest as nuclear puncta, exhibiting liquid-like properties 43.

Intriguingly, Pol II exhibits colocalization with mediators within stable condensates while diligently executing the transcription of both messenger RNAs and various non-coding RNAs, all of which display hallmarks of phase-separated condensates (e.g., rapid fluorescence recovery post-photobleaching and susceptibility to 1,6-hexanediol, an inhibitor of LLPS) 46, 47. Furthermore, Pol II initiates cluster formation by interacting with the intrinsically disordered carboxy-terminal domain and activators, and the subsequent phosphorylation of this domain leads to its release from the clusters, thereby triggering the transcription of the target gene 48. Recent investigations have unveiled that biomolecular condensation augments the recruitment of the negative elongation factor to promoters and activates the positive transcription elongation factor b, thereby facilitating Pol II elongation 49, 50. This orchestrated process orchestrates the transition from promoter-proximal pausing to transcription elongation 49, 50. Interestingly, the products of transcription, i.e., RNAs, are not mere passive spectators but actively partake in influencing the formation and characteristics of condensates through a feedback mechanism. Henninger et al. have illuminated this intricate interplay by revealing that transcriptional regulation incorporates a feedback loop wherein low levels of RNA serve as promoters of transcriptional condensate formation, whereas elevated RNA levels can dismantle transcription condensates, consequently downregulating transcription 51.

#### LLPS in DNA damage

DNA damage possesses the potential to instigate genomic instability, a prominent hallmark of cancerous cells. One of the initial cellular responses to DNA damage entails the enzymatic synthesis of poly(ADP-ribose) (PAR) polymers, orchestrated by the enzymes of PAR polymerase 1 52. The cellular rejoinder to DNA damage is notably characterized by the escalation of PAR levels, driven by the hyperactivation of PAR polymerase enzymes, which are proficient at discerning DNA breaks within the chromatin structure 53. Consequently, this instigates the swift accumulation of proteins bearing low-complexity sequence domains 53.

Remarkably, entities such as FUS/translocated in sarcoma, Ewing sarcoma (EWS), and TATA-box binding protein associated factor 15 emerge as prominent facilitators of phase separation, thus contributing significantly to this intricate landscape 54. The phenomenon of FUS-driven LLPS demonstrates remarkable efficacy in recruiting and sequestering splicing factors at sites of DNA damage 55. Furthermore, the phase separation of tumor protein p53 binding protein 1 (TP53BP1), a critical tumor suppressor protein intricately involved in orchestrating the balance between cell division and cell cycle arrest, imparts profound influence upon the fluid-like behavior governing DNA repair processes 56, 57.

Another compelling study underscores that the Rad52 DNA repair protein undergoes accumulation within distinct droplets, effectively collaborating with DNA damage-inducible intranuclear microtubule filaments. This collaboration enhances the aggregation of DNA damage sites, thereby contributing to the preservation of genomic stability 58. Additionally, the activity of cytoplasmic nucleic acid exonuclease three prime repair exonuclease 1 (EXO1) experiences modulation through the process of cyclic GMP-AMP synthase-driven phase separation, culminating in the formation of molecular condensates that effectively curtail DNA deterioration 59. Thus, a more profound understanding of the intricate mechanisms underpinning how LLPS regulates DNA repair holds substantial promise in unveiling novel therapeutic opportunities.

#### LLPS in chromatin organization

In most eukaryotes, a significant portion of the genome is ensconced within heterochromatin, a multifaceted scaffold pivotal for nuclear architecture, DNA repair, genome stability, and the quelling of transposons and gene expression 60, 61. It is posited that heterochromatin's gene-silencing activity arises, in part, from the capability of heterochromatin protein 1 (HP1), the “reader” of histone H3 lysine 9 methylation (H3K9me), to form complexes through increased multivalent interactions with H3K9me-modified chromatin 20. Considering heterochromatin organization from the perspective of phase separation provides a foundation for uncovering genomic conformational disturbances and their links to diseases. For example, methyl-CpG-binding protein 2 (MECP2), a chromatin organizer governing gene expression, competes with linker histone H1 to form distinct chromatin condensates in vitro and heterochromatin foci in vivo 62.

LLPS is intricately linked to chromatin modifications which can modulate phase-separation properties, thereby influencing chromatin organization and function. Post-translational modifications (PTMs) are well-established as pivotal regulators of biomolecular condensate formation 63.

Phosphorylation stands out as a highly characterized PTM that exerts plays a central role in signal transduction pathways 64. For instance, the activation of dual-specificity tyrosine-phosphorylation-regulated kinase 3 (DYRK3) facilitates the dissolution of SGs, releasing the mechanistic target of rapamycin complex 1 (mTOR1) for signaling and enhancing its activity by direct phosphorylation of the mTOR1 inhibitor 40-kDa proline-rich Akt substrate (PRAS40) 65. Intriguingly, phosphorylation promotes the pathological aggregation of microtubule-associated protein Tau by fostering electrostatic interactions conducive to phase separation, shedding light on the connection between LLPS and neurodegeneration in tauopathies 66.

Arginine methylation, a common PTM with broad implications across various cellular processes (e.g., chromatin remodeling, RNA processing, and cell signaling), directly interferes with phase separation by disrupting cation-π interactions, as exemplified in FUS 67.

Furthermore, small ubiquitin-like modifier (SUMOylation) represents an additional PTM that modifies cohorts of functionally related proteins through covalent attachment to lysine residues in a multitude of proteins 68. PML proteins, for instance, undergo extensive SUMOylation, subsequently recruiting client proteins into PML nuclear bodies through SUMO-interacting motifs/SUMO interactions 69.

#### LLPS in the permeation barrier

Extensive evidence suggests that multivalent protein assemblies are crucial for phase separation. Li et al. suggested a mechanism through which multivalent interactions could result in sharp transitions between physically and functionally distinct states and connect disparate length scales in the cell. This process potentially contributes to forming the structure and function of the cellular body 12. The primary pathway for nuclear-cytoplasmic communication is facilitated by nuclear pore complexes. These complexes are comprised of closely packed, inherently disordered nucleoporins rich in phenylalanine and glycine. They create a selective barrier that regulates permeability 70. These phenylalanine-glycine-rich nucleoporins form the core pore of the nuclear pore complexes and are instrumental in managing nucleocytoplasmic transport 71, 72. Their unique sequences allow them to undergo LLPS, forming hydrogel-like structures crucial for the regulation of cargo transport into and out of the nucleus.

### Involvement in enzymatic reactions

Evidence suggests that the net effect of crowding because of complex coacervation of multivalent macromolecules is to increase reaction rates. For example, LLPS may achieve precise control over the rate of enzymatic reactions by finely modulating the local concentration of reactants 73. Furthermore, LLPS compartmentalizes the mixed solution to produce a condensate with a particular material concentration., LLPS facilitates the formation of specific affinity interaction domains within cells, involving enzymes, substrates, and other biomolecules. This phenomenon contributes to the organized assembly and regulation of enzymatic reactions 74.

### Involvement in signal transduction

Intracellular signaling networks are essential for regulating cellular behavior and homeostasis. Recent research indicates that biomolecular condensates enhance the presence and control the function of signaling molecules through multivalent interactions 75, 76. These interactions, common in membrane-associated signaling pathways, suggest a broader applicability for such regulatory mechanisms 77.

LLPS is instrumental in the assembly of membrane receptors and associated signaling entities, evident in processes like T-cell receptor (TCR) signaling in immune cells and the activation of cyclic GMP-AMP synthase (cGAS) and stimulator of interferon genes 17, 78, 79. TCR phosphorylation, for instance, leads to the spontaneous formation of liquid-like clusters of downstream signaling proteins like GRB2, phospholipase C, and others, enhancing actin polymerization and contributing to TCR signaling 17. In the context of innate immunity, cGAS activation by cytosolic DNA triggers a phase transition, facilitating immune signaling 78. In the context of innate immunity, cGAS activation by cytosolic DNA triggers a phase transition, facilitating immune signaling. This activation results in a cascade of events, including the production of type I interferons and proinflammatory cytokines 80. Additionally, recent findings show that stimulator of interferon genes forms structured condensates, attracting TANK-binding kinase 1 (TBK1) and regulating innate immune responses 79. These results demonstrated that protein phase separation can create a distinct physical and biochemical compartment that facilitates signaling.

### Involvement in stress response to external stimuli

Cellular adaptation mechanisms to diverse physicochemical environmental conditions (like temperature, pH, salinity, redox states) involve the dynamic formation or dissociation of biomolecular condensates under stress. In response to abrupt environmental changes, these condensates can rapidly assemble or disperse.

During heat shock, cells compartmentalize protein aggregates, impacting mRNA expression under stress 81. Adverse conditions can also trigger the formation of SGs, which safeguard cells against various stressors 82. For instance, under heat stress, SGs containing mRNA and proteins influence mRNA localization, translation, and degradation, as well as regulate signaling pathways and antiviral responses 35. This dynamic aggregation and disaggregation of proteins is an adaptive, self-regulating process that may enhance organismal resilience during stress.

Zlotorynski et al. demonstrated that changes in pH can influence phase separation. The yeast prion protein Sup35 forms liquid-like condensates at pH 6, which solidify into a gel-like state and dissolve upon pH increase, aiding in translation resumption and enhancing cellular fitness post-stress 36, 83.

Furthermore, salt concentration and charge variations can induce cellular stress. Cummings et al. observed that green fluorescent protein undergoes LLPS more readily at higher salt concentrations, forming liquid condensates from solid precipitates 37. Also, macromolecular crowding under hyperosmotic stress leads to the formation of liquid-demixing condensates of apoptosis signal-regulating kinase 3 (ASK3), influencing its activity 84. PolyA binding protein-binding protein 1 (Pbp1), a regulator of target of rapamycin complex 1 (TORC1) signaling and autophagy, interacts with TORC1 during respiratory growth, triggering TORC1-mediated autophagy. This process is crucial for eliminating SGs and damaged organelles, thus protecting cells from oxidative stress due to redox imbalances 85, 86.

## LLPS contributes to the development of cancer

LLPS has been implicated in the epigenetic dysregulation that contributes to carcinogenesis and tumor progression 87-89. Recent studies suggest that phase-separated condensates may play a role in modulating drug distribution and concentration, thereby affecting drug efficacy and resistance in cancer cells 90. Table 1 presents an overview of potential targets and emerging strategies for treating malignancies linked to LLPS, offering a theoretical framework for the identification of effective target molecules.

### LLPS in prostate cancer

Prostate cancer, a prevalent malignancy in men worldwide, is effectively treated in its early stages with surgery or radiation, often combined with androgen-deprivation therapy in more advanced cases 91, 92. However, the response to this treatment varies, commonly leading to castration-resistant prostate cancer (CRPC), a lethal stage of the disease 93. Addressing the resistance to antiandrogen therapies in CRPC patients is a critical aspect of ongoing clinical research 94.

Recent studies have identified the tumor suppressor speckle-type POZ protein (SPOP) as frequently mutated in solid tumors, particularly prostate cancer. These mutations disrupt phase separation and co-localization in membraneless organelles 95. Notably, prostate cancer-associated SPOP mutations have been found to enhance autophagy and activate the NFE2L2/NRF2 pathway, crucial in managing oxidative stress, by directly modulating sequestosome1 (SQSTM1) LLPS and ubiquitination 96. This finding supports the possibility that this oncogenic pathway may guide targeted therapy toward SPOP-mutated cancers. A recent study showed that androgen receptor (AR) forms dynamic AR-rich, liquid-like foci with coactivator MED1 to SEs in cellular prostate cancer models, promoting an oncogenic transcriptional program 97. This discovery suggests that targeting the SPOP mutation could be a promising therapeutic approach. In cellular models of prostate cancer, the AR forms dynamic, liquid-like foci with MED1 in SEs, driving an oncogenic transcriptional program 96. OCT4 recruitment to specific genomic loci activates other TFs at SEs, indicating that disrupting these interactions could be a novel therapeutic strategy for advanced tumors 98.

Xie et al. discovered that the AR LLPS inhibitor ET516 potentially disrupts the AR feedback mechanism. This compound acts as a precursor to increased resistance by specifically targeting AR LLPS, countering resistance mechanisms arising from AR mutations or splicing events 94. Additionally, UT-143, an AR-selective irreversible covalent antagonist, was found to hinder LLPS formation and mutagenesis, leading to chromatin condensation and disassembly of the AR splice variant 7 (AR-V7) interactome, resulting in a transcriptionally inactive complex 99. Lastly, research has shown that lysine-specific demethylase 1 (LSD1), an AR coactivator in prostate cancer, drives disease progression through SE-mediated oncogenic programs, which could be countered using LSD1 inhibitors to suppress CRPC growth 100.

### LLPS in osteosarcoma

Osteosarcoma, a highly aggressive primary bone malignancy prevalent in pediatric and adolescent populations, is frequently associated with unfavorable prognosis and low survival rates, particularly in cases involving metastasis and recurrent disease 101. Substantial evidence from prior investigations underscores the crucial role of the *MYC*-driven SE signaling pathway in osteosarcoma tumorigenesis 102. The administration of SE inhibitors, such as THZ1 and JQ1, has demonstrated marked success in abrogating the proliferative, migratory, and invasive capacities of osteosarcoma cells. Thus, the therapeutic targeting of the* MYC*/SE axis emerges as a promising avenue for the management of osteosarcoma patients 102.

In a study conducted by Lu et al., it was elucidated that core regulatory circuit components, namely homeobox B8 (HOXB8) and fos-like antigen 1 (FOSL1), residing proximal to the SE locus in osteosarcoma, exhibit the ability to form dense and dynamically phase-separated droplets in vitro and liquid-like puncta within cell nuclei. These observations suggest a potential role in supporting SE-driven transcriptional processes 103. Furthermore, the utilization of the histone H3 lysine 27 (H3K27) demethylase inhibitor GSK‐J4 disrupts the phase separation dynamics of core regulatory circuit factors. This disruption results in reduced chromatin accessibility within SE regions and the subsequent inhibition of aberrant oncogenic transcriptional programs. As such, GSK‐J4 emerges as a prospective therapeutic candidate for patients afflicted with metastatic and chemoresistant osteosarcoma, offering promising prospects for improving clinical outcomes 103.

### LLPS in multiple myeloma

Multiple myeloma progression is linked to genetic and epigenetic anomalies 104. Recent studies highlight the impact of altered epigenetic mechanisms in these cancer cells, leading to immature, drug-resistant phenotypes 105. Notably, steroid receptor coactivator-3 (SRC-3), characterized by its LLPS properties, is integral to drug resistance in multiple myeloma 106. Research indicates that SRC-3 LLPS enhances histone methyltransferase nuclear receptor binding SET domain protein 2 (NSD2) recruitment, influencing histone H3 at lysine 36 (H3K36me) and apoptosis, thereby facilitating drug resistance. Furthermore, the SRC-3 inhibitor SI-2 shows potential in sensitizing cells to bortezomib by disrupting NSD2 106. Regulating gene expression to decrease LLPS may increase drug efficacy, and targeting SRC-3 could reduce bortezomib resistance. Additionally, small-molecule bromodomain inhibitors like JQ1 in xenograft multiple myeloma models demonstrate significant antiproliferative effects, indicating bromodomain and extra-terminal domain (BET) inhibition as a promising cancer treatment strategy 107.

### LLPS in lung cancer

Lung cancer, notably with a high incidence and mortality rate, is a leading cause of cancer deaths 108. Despite advances, resistance to therapy remains a challenge in advanced non-small cell lung cancer (NSCLC) 109. A significant contributor to NSCLC oncogenesis is the mesenchymal lymphoma kinase (anaplastic lymphoma receptor tyrosine kinase [ALK]) fusion mutations, often associated with accelerated cancer progression 110. Qin et al. identified that the echinoderm microtubule-associated protein-like 4/ALK (EML/ALK) variant undergoes phase separation in tumors, enhancing STAT3 phosphorylation and tumor transformation 111. Additionally, long non-coding RNA (lncRNA) MNX1-AS1, upregulated by gene amplification and c-Myc, stabilizes TF1 mRNA in association with insulin-like growth factor 2 mRNA binding protein 1 (IGF2BP1) by LLPS, exhibiting potent anti-tumor effects in xenograft models, thus presenting a potential biomarker and therapeutic target 112. Zhang et al. observed that myristoylation induces phase-separated liquid droplets of enhancer of zeste homolog 2 (EZH2) in lung cancer cells, compartmentalizing and activating STAT3, and promoting cell growth 113. Targeting EZH2 myristoylation emerges as a novel lung cancer treatment strategy.

Further, steroid receptor coactivator-1 (SRC-1) forms phase-separated condensates with Yes-associated protein/TEA domain, enhancing gene expression. This process is disruptible by elvitegravir, an anti-HIV drug, inhibiting YAP's oncogenic activity 114. Lu et al. demonstrated that lncRNA MELTF-AS1 binds to and induces phase separation of Y-box binding protein 1 (YBX1), activating ANXA8 transcription and NSCLC tumorigenesis 115. Moreover, ubiquitin-specific peptidase 42 (USP42) regulates phase separation of spliceosome component PLRG1, facilitating mRNA splicing and tumorigenesis 116. Treatment with BRD4-targeting JQ1, combined with chemoradiotherapy and anti-PD-L1 antibody, enhances anti-tumor immunity in NSCLC 117.

### LLPS in breast cancer

Breast cancer ranks as the second most prevalent cancer among women globally, with significant mortality implications. Despite advancements in screening and treatment, high rates of invasion and metastasis contribute to low survival rates 118. Research shows that tumor suppressor SPOP mutants, by impairing phase separation, can induce breast cancer through mutation-caused mislocalization 95. In the cellular context, ATP, acting as an estrogen cofactor, is involved in the dynamic phase separation process, influencing the pathophysiology of breast cancer 119, 120. Nudix hydrolase 5 (NUDT5), crucial for ATP synthesis in breast cancer cell nuclei, is targeted by TH5427, a specific inhibitor that disrupts nuclear ATP generation, impacting hormone-induced chromatin remodeling and progestin-dependent gene regulation 121. Studies using a mouse breast cancer model have highlighted that cationic polymers, like cDex and DETA-Dex, can impede RNA LLPS, thereby inhibiting TGFβ1 mRNA translation in tumor cells 122. Moreover, Hu et al. identified that the cell-polarity protein Par3 facilitates the phase separation of junctional adhesion molecule-A during breast cancer metastasis, altering osmotic pressure and mechanical properties 123. Inhibiting atypical protein kinase C (aPKC) can reduce Par3's mechanical transmission, thus hindering breast cancer invasion, migration, and potentially improving survival 123. Additionally, drug resistance in breast cancer is closely linked to cancer stem cells. The phase separation of TAZ-Nanog homeobox enhances the transcription of SOX2 and OCT4. Targeting Nanog homeobox or TAZ could therefore elevate chemosensitivity by diminishing cancer stem cell prevalence in breast cancer cells 124.

### LLPS in pancreatic cancer

Pancreatic cancer, characterized by its aggressive nature and ranking foremost among asymptomatic malignancies, exhibits a dismal prognosis, with significant survival improvement only achievable through resection achieving macroscopic tumor clearance 125. Consequently, precision medicine emerges as crucial for enhancing therapeutic outcomes and prognoses for this disease. Notably, 1,6-hexanediol, by disrupting protein-mediated abnormal LLPS, substantially reduces MYC expression, effectively curtailing pancreatic cancer growth 126. The serine/arginine-rich protein kinase 2 (SRPK2), identified as a key regulator of stress granule (SG) formation in obesity-associated pancreatic ductal adenocarcinoma, modulates SG assembly through the hyperactivation of the IGF1/PI3K/mTOR1/S6K1 pathway. Furthermore, SRPK2, as a substrate of S6K1, is implicated in mRNA stability, splicing, and lipid metabolism 127, 128. The S6K1 inhibitor PF4708671 shows preferential efficacy in diminishing SGs and inhibiting growth in obesity-related pancreatic cancer 127. Additionally, the low-complexity sequence domains of histone lysine (K)-specific methyltransferase 2D (KMT2D) facilitate a LLPS environment, which is instrumental in regulating histone monomethylation and transcription, thus promoting pancreatic cancer progression 129. Therefore, targeting the LLPS microenvironment emerges as a promising strategy in pancreatic cancer therapy.

### LLPS in leukemia

Leukemia is a malignant hematologic cancer with a long history of targeted therapy development. TF Yin-Yang 1 (YY1) is a new target for treating acute myeloid leukemia. It binds to histone deacetylase 1/3 (HDAC1/3) and controls the high production of methyltransferase-like protein 3 (METTL3) through moderate LLPS 130. Treatment with an HDAC inhibitor treatment significantly reduces the binding of YY1 to HDAC 1/3, resulting in an excessive LLPS state, thereby reducing the expression of METTL3 and the proliferation of acute myeloid leukemia cells 130. RNA-binding protein YTH N6-methyladenosine RNA binding protein C1 (YTHDC1) is highly expressed in many cases of acute myeloid leukemia and is a new potential target for treating this disease 131. Cheng et al. reported that YTHDC1 binds m6A-mRNA by phase separation to form nuclear YTHDC1-m6A condensates, which prevent the degradation of target gene mRNA, thereby allowing acute myelocytic leukemia cells to survive and maintain their undifferentiated state 132.

A study revealed that deneddylation of PML /retinoic acid receptor α restores phase separation to reconstitute functional PML nuclear bodies and activates retinoic acid receptor α, the eradication of acute promyelocytic leukemia. Hence, PML proteins in leukemia are promising targets 133, 134. In addition, it was found that nucleoporin 98 (NUP98) fusion oncoproteins drive LLPS with the help of homo- and hetero-typic interactions, thereby forming aberrant nuclear loci and affecting gene expression in leukemia 135. Therefore, therapeutic modulation of aberrant LLPS using fusion oncoproteins is a compelling prospect for cancer treatment. In T-cell acute lymphoblastic leukemia, heterozygous somatic mutations of T-cell acute lymphocytic leukemia protein 1 (TAL1) oncogene are acquired, which introduce binding motifs for the TF myeloblastosis oncogene and creates a SE upstream of the TAL1 oncogene. In this way, the aberrantly formed SE drives monoallelic TAL1 expression, promoting the progression of leukemia 136.

Ubiquitously transcribed tetratricopeptide repeat, X chromosome (UTX) (a critical tumor suppressor) regulates genome-wide histone modifications and high-order chromatin interactions in a condensation-dependent manner 137. UTX encodes the H3K27 demethylase; thus, GSK-J4 is a novel, highly promising epigenetic therapy option against TAL1-positive T-cell acute lymphoblastic leukaemia 138.

### LLPS in sarcoma

The therapeutic targeting of translocation-driven sarcomas presents significant challenges. Recent focus on fusion proteins has opened avenues for identifying novel therapeutic targets, exemplified by FUS-C/EBP HOmologous protein (FUS-CHOP) in myxoid liposarcoma and EWS RNA-binding protein 1/friend leukemia virus integration 1 (EWSR1/FLI1) in Ewing's sarcoma 139, 140. FUS-CHOP is observed to form phase-separated condensates that co-localize with BRD4 within SEs, impacting chromatin remodeling and transcription, thus offering a potential therapeutic avenue for myxoid liposarcoma 141. The prion-like domain in EWS-FLI1 is associated with aberrant phase separation events, leading to the activation of numerous gene targets in Ewing's sarcoma 139. Hsp104, a hexameric AAA+ protein disaggregase from yeast, demonstrates efficacy in mitigating the toxicity and aggregation of FUS-CHOP and EWS-FLI, suggesting its potential utility in counteracting the deleterious effects of abnormal fusion proteins in sarcomas 142. Additionally, the oncoprotein SS18-SSX, a distinctive marker of synovial sarcoma, has been scrutinized by Chang et al., who identified that the self-association of its intrinsically disordered QPGY domain leads to LLPS, contributing to its oncogenic activity in synovial sarcoma 143. Consequently, targeting phase-separated structures of SS18-SSX1 with small molecules emerges as a promising therapeutic strategy for synovial sarcoma.

### LLPS in glioblastoma

Glioblastoma, the most lethal form of primary brain cancer, presents a formidable clinical challenge. Current treatment strategies, encompassing surgery, chemotherapy, and adjuvant radiotherapy, yield a median survival duration of merely 18 months for afflicted patients 144. Research led by Wei et al. has highlighted the critical role of non-POU-domain-containing octamer-binding protein (NONO), a TAZ‐binding protein, in facilitating the TAZ-driven oncogenic transcriptional mechanism and promoting TAZ-mediated LLPS, thereby driving the transcriptional program of glioblastoma 145. The suppression of NONO expression has been shown to inhibit TAZ-driven tumorigenesis, suggesting the modulation of NONO as a novel therapeutic approach against TAZ-driven glioblastoma 145. Furthermore, Gene Set Enrichment Analysis has identified a strong association of fatty acid-binding protein 5 (FABP5), implicated in LLPS, with key signaling pathways in glioblastoma, including nuclear factor-kappa B (NF-κB) signaling 146, 147. A negative correlation between BRD4 expression and overall survival in glioma patients has been observed, with the novel BRD4 inhibitor GNE987 targeting c-Myc expression and modulating transcription through BRD4 downregulation 148. Additionally, recent studies have unveiled that the nucleoprotein AHNAK impedes TP53BP1 oligomerization and phase separation, thereby curtailing glioma cell proliferation and inducing apoptosis 56, 149.

### LLPS in ovarian cancer

Ovarian cancer remains one of the leading causes of death in women worldwide, although the prognosis of early-stage disease is good 150. Research indicates that genes related to LLPS are aberrantly expressed in epithelial ovarian cancer, influencing the cell cycle and DNA replication, and thus presenting as potential prognostic indicators for this malignancy 151. Despite the theoretical efficacy of BET bromodomain inhibitors in the treatment of epithelial ovarian cancer, initial clinical trials have yielded suboptimal results 152. Zhang et al. have suggested a novel approach, wherein the combined use of phase separation of BRD4 droplets and aurora kinase inhibitors effectively counteracts the antagonistic impact of BET bromodomain inhibitors, thereby targeting and eradicating JQ1-resistant ovarian cancer cells 153.

### LLPS in esophageal cancer

Esophageal squamous cell carcinoma, classified among the deadliest forms of human cancer, is notorious for its treatment resistance and high recurrence rate, with a notable absence of approved targeted therapies currently available 154. In this context, the macrophage-associated lncRNA (*MALR*) is significantly overexpressed in esophageal squamous cell carcinoma. It is hypothesized that *MALR* interacts with the double-stranded RNA-binding domain 3 of interleukin-enhancing factor 1 (ILF3), consequently inducing ILF3 LLPS and activating hypoxia-inducible factor 1α (HIF1α) signaling pathways 155. Therefore, the MALR-ILF3-HIF1α axis presents itself as a potential therapeutic target in the treatment of esophageal squamous cell carcinoma.

### LLPS in colorectal cancer

Colorectal cancer ranks as one of the most fatal diseases globally, with patients frequently facing high recurrence, metastasis, diagnostic challenges, and poor prognostic outcomes 156. In colorectal cancer tissues, the expression of NONO is notably elevated. NONO-mediated LLPS recruits DNA-dependent protein kinase and nuclear epidermal growth factor receptor, enhancing their phosphorylation and consequently accelerating DNA repair in tumor cells 157. Kondo et al. identified a novel compound, aminocyclopropenone 1n (ACP-1n), that impedes SE-driven MYC expression by inhibiting BRD4 functionality in the nucleus of colorectal cancer HCT4 cells in vitro, thereby suppressing oncogenic transcription 158. Additionally, the nucleolar protein53 (Nop53) is overexpressed in colorectal cancer and correlates with poor prognosis. Nop53 forms cohesions in the nucleolus and exhibits sensitivity to 1,6-hexanediol treatment 159. It inhibits *p53* activation and enhances radioresistance in colorectal cancer cells, making it a potential target for increasing radiosensitivity in cancer patients 159. Moreover, DExD-box helicase 21 (DDX21), a notable RNA-binding protein, is highly expressed in colorectal cancer. The phase-separated condensates of DDX21 target and activate minichromosome maintenance complex component 5 (MCM5), facilitating colorectal cancer cell migration and invasion, and triggering the activation of the Epithelial-mesenchymal transition pathway, which is crucial in the metastatic regulatory circuitry 160.

### LLPS in Wilms tumor

Nephroblastoma is one of the most common solid tumors in children; particularly, Wilms tumor is the most common type of pediatric kidney tumors 161. Within this context, AF9 and ENL, both YEATS domain-containing proteins and readers of histone acetylation, play pivotal roles in chromatin modification and transcription regulation 162, 163. Furthermore, mutations in the YEATS domain of ENL have been identified as functionally significant in Wilms tumor. These mutations augment phase separation and transcription, leading to an aberrant gene expression profile 164. This emerging understanding offers novel insights into potential mechanism-based strategies to mitigate the oncogenic impacts of ENL mutations.

### LLPS in liver cancer

The liver ranks as the sixth most common primary cancer site in humans, with liver cancer patients often facing low survival rates unless the disease is detected early 165. A circular RNA known as *VAMP3* has been identified to facilitate the phase separation of cytoplasmic activation/proliferation-associated protein 1 (CAPRIN1), thereby promoting stress granule formation in cells. This process, in turn, inhibits c-*Myc* translation, regulating cell proliferation and metastasis 166. Furthermore, glycogen accumulation has been recognized as a critical oncogenic initiator in malignant liver transformation. This accumulation disrupts Hippo signaling through glycogen phase separation, thereby exacerbating tumor development 167.

## Regulatory ethods of applying LLPS in cancer treatment

Given that LLPS can impact tumorigenesis through various pathways, there is a need to develop practical strategies for modulating the formation of condensates and targeting cancer-associated proteins. Recent advancements have revealed several regulatory methods for biomolecular condensates, including molecule drug treatment, RNA interference, and DNA editing.

**Figure 3:**
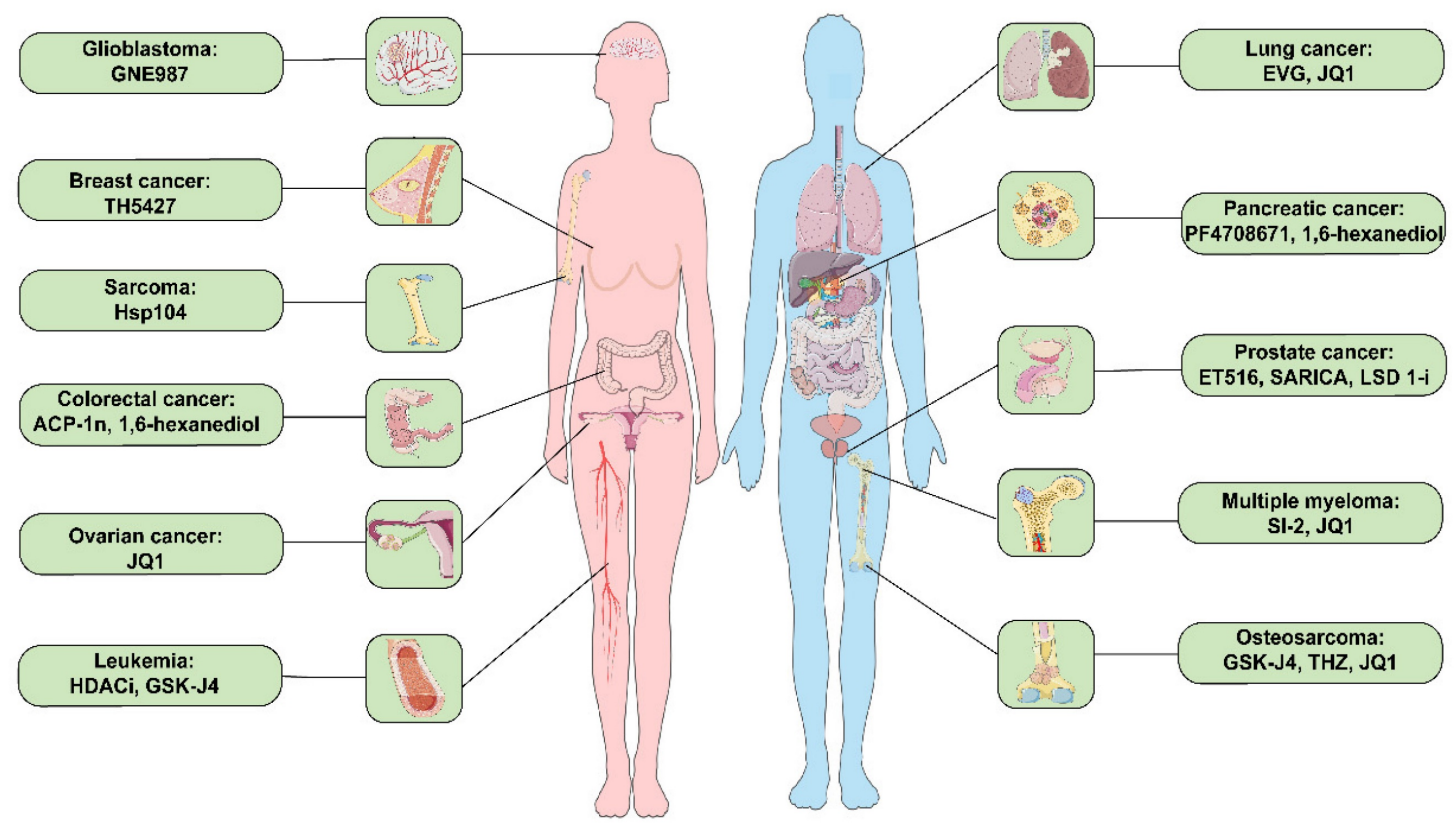
Small molecule inhibitors related to LLPS in prevalent clinical malignancies. These diseases include glioblastoma, breast cancer, sarcoma, colorectal cancer, ovarian cancer, leukemia, lung cancer, pancreatic cancer, prostate cancer, multiple myeloma, and osteosarcoma cancer. ACP-1n, aminocyclopropenone 1n; EVG, elvitegravir; HDACi, histone deacetylase inhibitor; LSD 1i, lysine-specific histone demethylase 1 inhibitor; SARICA, small-molecule-selective AR-irreversible covalent antagonists.

As anticipated, evidence suggests that small molecule drug treatment can effectively modulate phase separation (**Table 2**). For instance, 1,6-Hexanediol disrupts hydrophobic interactions, thereby compromising the 3-dimensional genome organization in living cells and inhibiting LLPS 47. JQ1 inhibits osteosarcoma, multiple myeloma, and ovarian cancer cell proliferation, migration, and invasion by suppressing SEs genes as inhibitors targeting transcriptional activators (**Figure 3**) 102, 107, 153. Additionally, HDACi (NCT03564704) and TSA (NCT03185429) are currently being evaluated in clinical trials to determine their efficacy and safety in treating patients with leukemia and esophageal tumors.

RNA interference to modulate LLPS can be a potential regulatory method. RNA interference relies on the cell creating small RNA molecules that can target and inhibit the harmful RNA sequences that need to be silenced 168. For example, the suppressor of gene silencing 3 (SGS3) LLPS drives RNA-dependent RNA polynerase 6 (RDR6) to form small interfering RNA bodies in cytoplasm, which is essential for small interfering RNA production and gene silencing 169.

Furthermore, the gene editing technique known as clustered regularly interspaced short palindromic repeat (CRISPR)-CRISPR associated (Cas) nuclease 9 (CRISPR-Cas9) offers another avenue for LLPS regulation. This technique is derived from the natural defense system of bacteria and archaea against viral invasion 170. Notably, Shin et al. recently reported CasDrop, a novel optogenetic technology developed based on CRISPR-Cas9, enables controlled liquid condensation at specific genomic loci 171.

## Conclusions

Over the past decade, LLPS has emerged as a burgeoning and captivating concept in cellular biology. Despite its nascent stage, research in phase separation holds considerable potential. Cancer, often associated with dire prognoses and challenging treatments, also incurs substantial economic burdens on individuals and societies. The selective drug distribution and concentration abilities within phase-separated structures can influence drug efficacy and resistance in cancer cells. For example, Mitrea et al. have described a previously unexplored drug discovery approach based on identifying condensate-modifying therapeutics 172. Consequently, targeting the regulation of phase separation is becoming pivotal in both the diagnosis and treatment of diseases. Clinical trials exploring inhibitors that target transcriptional activators have reported reductions in tumor growth, underlining their therapeutic potential.

However, the understanding of LLPS in cancer remains incomplete. The direct link between the anticancer activity of transcription-related inhibitors and the regulation of biomolecular cohesions is not fully established. This gap in knowledge indicates the need for more comprehensive research. Moreover, most existing studies rely on cultured cancer cells, leaving the comparability of phase separation phenomena and biomolecular cohesions *in vitro* versus *in vivo* environments uncertain. Such differences cast doubt on whether current research findings can be translated into clinical settings. Additionally, the intercommunication mechanisms among these biomolecular condensates have yet to be elucidated. To address these critical issues, a multidisciplinary approach is essential. Molecular biologists and biochemists, for instance, are focusing on understanding the molecular mechanisms underlying LLPS. They study how specific proteins and nucleic acids interact to form phase-separated structures and how these structures influence cellular functions in cancer. Moreover, computational biologists play a crucial role in simulating these complex interactions and predicting how changes in LLPS could lead to cancerous states. They use computational tools to simulate the LLPS process, providing insights to guide experimental design and hypothesis testing. Clinical researchers and oncologists translate these findings into potential therapeutic strategies. Finally, this collaboration extends to chemists and drug developers, who leverage knowledge from these studies to design and synthesize new compounds that can modulate LLPS in cancer cells.

Furthermore, the predominant use of fluorescence recovery after photobleaching (FRAP) in previous studies to demonstrate LLPS has faced skepticism 173. The critics, including Thompson et al. 174, believe that FRAP relies on morphological assessment rather than quantitative data, which limits the study of LLPS. This critique underscores the urgent need for “new tools” to yield more precise data. Herein, we propose that by integrating techniques such as FRAP, Förster Resonance Energy Transfer, and super-resolution microscopy, researchers can gain a more comprehensive understanding of LLPS phenomena in cancer cells. These methods can provide quantitative data and deeper insights into the molecular interactions within phase-separated structures. We look forward to ongoing research that integrates multiple technologies, as it is expected to further clarify the relationship between cancer and LLPS potentially leading to breakthroughs in cancer treatment.

## Figures and Tables

**Table 1:** Transcription factors activated by LLPS and possible potential targets.

Tumor model	Complex	Cellular mechanisms (Transcription factors)	Reference
Prostate Cancer	SPOP	SPOP mutations enhance autophagy and NFE2L2 activation by directly modulating SQSTM1 LLPS and ubiquitination.	96
	MED1	MED1 is required for androgen receptor-mediated transcription.	97
	OCT4	OCT4 is recruited to specific genomic loci to activate other TFs.	98
	AR-SV	AR-SVs promote tumor growth by orchestrating transcriptional reprogramming.	99
	LSD1	LSD1 function as a transcriptional corepressor and coactivator.	100
	BRD4	BRD4 participates in super-enhancers organization and oncogenes expression regulation.	175
	rIGSRNA	Tumor growth repression.	176
Osteosarcoma	MYC	MYC regulates the SEs containing genes and mediates the transcriptional amplification of its target genes.	102
	HOXB8 and FOSL1	HOXB8 and FOSL1 form condensates to regulate chromatin accessibility and oncogenic transcription.	103
Multiple Myeloma	SRC-3	SRC-3 LLPS enhanced recruitment of NSD2 to the condensate.	106
	BRD4	BRD4 participates in super-enhancers organization and oncogenes expression regulation.	107
Lung Cancer	EML4-ALK	EML4-ALK LLPS activate STAT3 phosphorylation and downstream pathways of tumor transformation.	111
	MNX1-AS1	MNX1-AS1 binds to IGF2BP1 and drives its phase separation to maintain mRNA stability of c-Myc and E2F1	112
	EZH2	Myristoylation-mediated LLPS of EZH2 activates STAT3 signaling.	113
	YAP/TAZ	YAP/TAZ activates target genes and is directly involved in the control of S-phase entry and mitosis.	114
	MELTF-AS1	MELTF-AS1 regulates tumorigenesis by driving phase separation of YBX1 to activate ANXA8.	115
	USP42	USP42 controls phase separation of spliceosome component PLRG1.	116
Breast Cancer	NUDT5	NUDT5 affects the phase separation process by regulating ATP synthesis in the nucleus of breast cancer cells.	177
	Cationic polymers	Blocks the translation of TGFβ1 mRNA in tumor cells by inducing RNA LLPS.	122
	Par3	Par3 acts as a mechanical mediator of breast cancer aggressiveness.	123
	TAZ-NANOG	TAZ-NANOG LLPS promotes the transcription of SOX2 and OCT4.	124
Pancreatic Cancer	SRPK2	SRPK2 mediates SG formation through overactivation driven by the IGF1/PI3K/mTOR/S6K1 pathway.	127
	KMT2D	KMT2S low-complexity domains creates an LLPS environment that helps stabilize WDR5 protein and complex formation.	129
Leukemia	YY1	YY1 binds to HDAC1 / 3 and regulates the high expression of METTL3.	130
	YTHDC1	YTHDC1 binds to m6A-mRNA to form nYACs, protecting the stability of its target gene mRNA from degradation.	132
	PML/RARα	Deneddylation of PML/RARα restores its phase separation process to reconstruct functional nuclear bodies and activates RARα signaling	133
	NUP98	NUP98 results in the transcriptional activation of leukemogenic genes.	135
Myxoid liposarcoma	FUS-CHOP	FUS-CHOP recruits BRD4 to carcinogenic condensates.	141
Ewing sarcoma	EWS-FLI1	EWS-FLI1 binds to the protein chaperone network and regulates the transcription gene targets.	139
Synovial sarcoma	SS18-SSX	SS18 enriches BRG1 into the condensate through the interaction between SS1 and BRG18.	143
Glioblastoma	BYSL	BYSL is involved in the malignant progression of glioblastoma through the GSK-3β/β-linked protein signaling pathway.	178
	NONO	NONO depletion reduces nuclear TAZ LLPS, while ectopic NONO expression promotes the LLPS.	145
Ovarian cancer	BRD4	BRD4 participates in super-enhancers organization and oncogenes expression regulation.	152
Esophageal Cancer	ILF3	ILF3 encodes two double-stranded RNA -binding proteins to regulate gene transcription and protein translation.	155
	BRD4	BRD4 participates in super-enhancers organization and oncogenes expression regulation.	179
Colorectal cancer	NONO	NONO LLPS enhances DNA damage repair by accelerating nuclear EGFR-induced DNA-PK activation.	157
	BRD4	BRD4 participates in super-enhancers organization and oncogenes expression regulation	158
	NOP53	NOP53 inhibits p53 activation and enhances radio-resistance in colorectal cancer cells	159

**Table 2:** Small molecule inhibitors related to LLPS in malignant tumors and their mechanisms of action.

Tumor model	Inhibitors	Mechanisms	Reference
Prostate Cancer	ET516	ET516 blocks AR transcription and inhibits the growth of castration-resistant prostate cancer.	94
	SARICA	Interfering with LLPS formation or mutagenesis resulted in chromatin condensation and dissociation of the AR-V7 interactome.	99
	LSD 1-i	Target multiple oncogenic pathways in CRPC and that disrupting MYC signaling.	100
Osteosarcoma	THZ	THZ inhibits osteosarcoma cell proliferation, migration, and invasion by suppressing SEs genes.	102
	JQ1	JQ1 inhibits osteosarcoma cell proliferation, migration, and invasion by suppressing SEs genes.	102
	GSK-J4	GSK-J4 binds directly to HOXB8-IDR and breaks the loop CRC condensate.	103
Multiple Myeloma	SI-2	SI-2 disrupts SRC-3 LLPS and sensitizes Bortezomib treatment in myeloma cells.	106
	JQ1	JQ1 preferentially disrupts the transcription process.	107
Lung Cancer	EVG	regulates YAP transcriptional activity by reducing H3K27ac mark levels at YAP target genes	114
Breast Cancer	TH5427	TH5427 blocked nuclear ATP synthesis, chromatin remodeling, and gene regulation.	121
Pancreatic Cancer	1,6-hexanediol	disrupt protein-mediated abnormal LLPS and significantly reduce *MYC* expression.	126
	PF-4708671	S6K1 inhibition selectively attenuates SGs and impairs obesity-associated pancreatic ductal adenocarcinoma development.	127
Leukemia	HDACi	HDACi inhibits METTL3 expression and proliferation of acute myeloid leukemia cells.	130
Glioblastoma	1-AZ	1-AZ inhibition of GSK-1β partially reverses *BYSL* down-regulation of β-linked protein transcriptional activity.	178
Ovarian cancer	JQ1	JQ1 preferentially disrupts the transcription process.	153
Esophageal Cancer	TSA	TSA promotes esophageal squamous cell carcinoma migration and Epithelial-mesenchymal-transition through two signaling pathways of BRD4/ERK1/2.	179
Colorectal cancer	ACP-1n	ACP-1n blocks BRD4 in the nucleus of colorectal cancer HCT4 cells to suppress SE-driven *MYC* expression.	158
